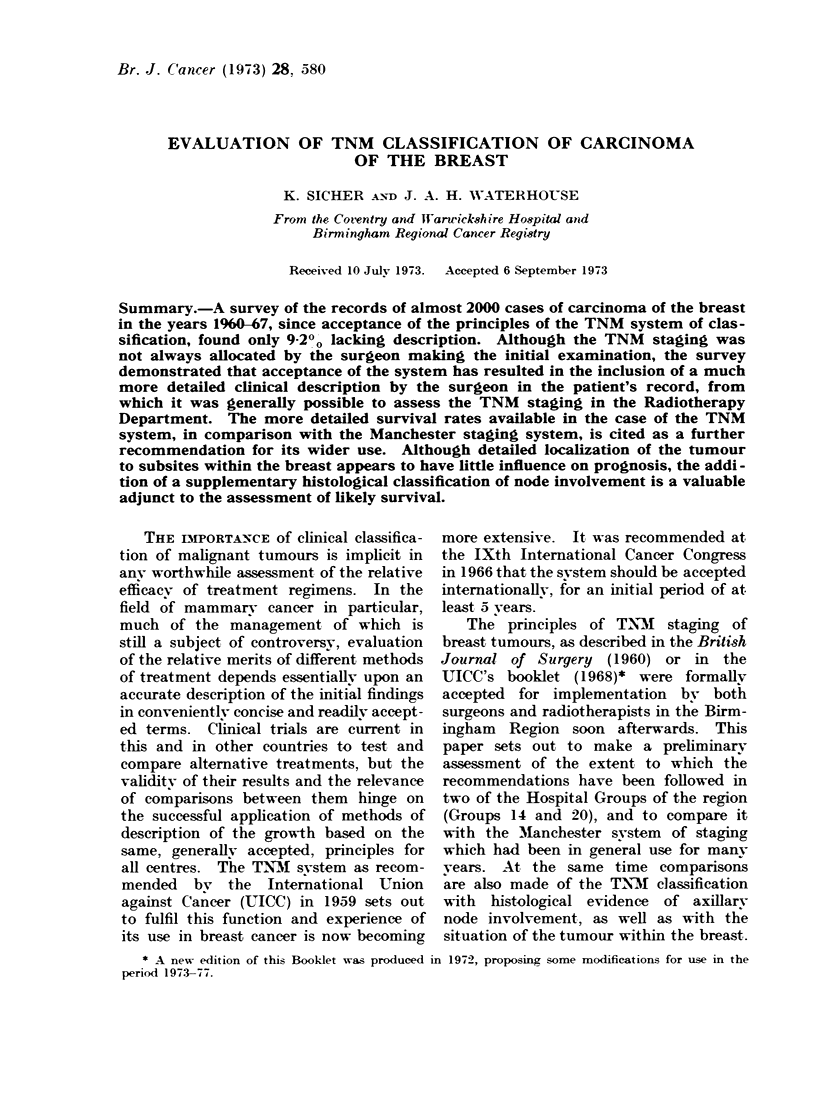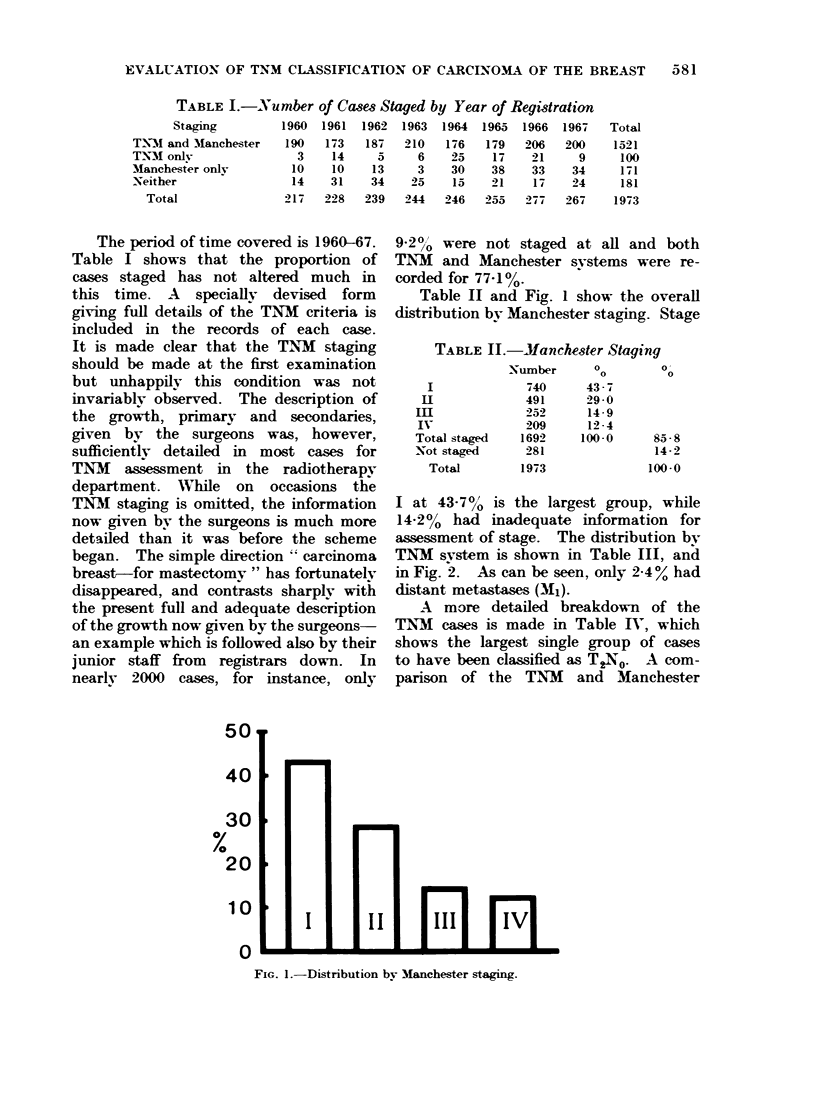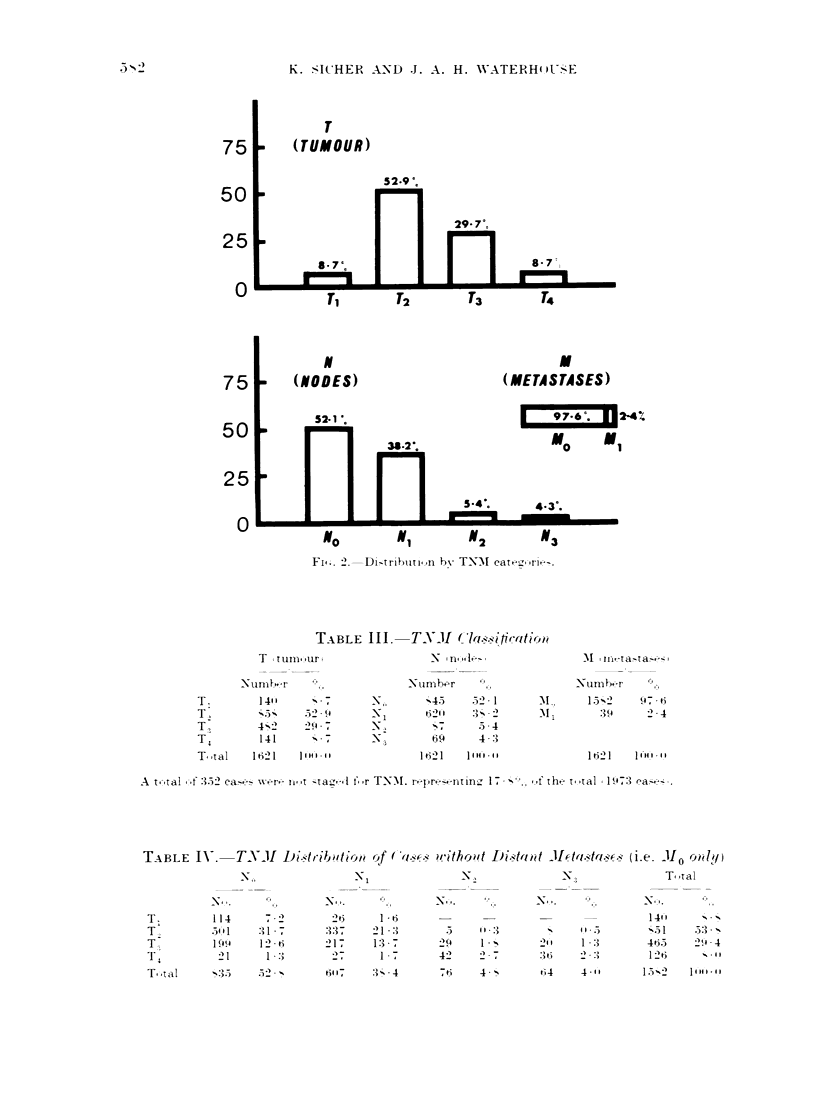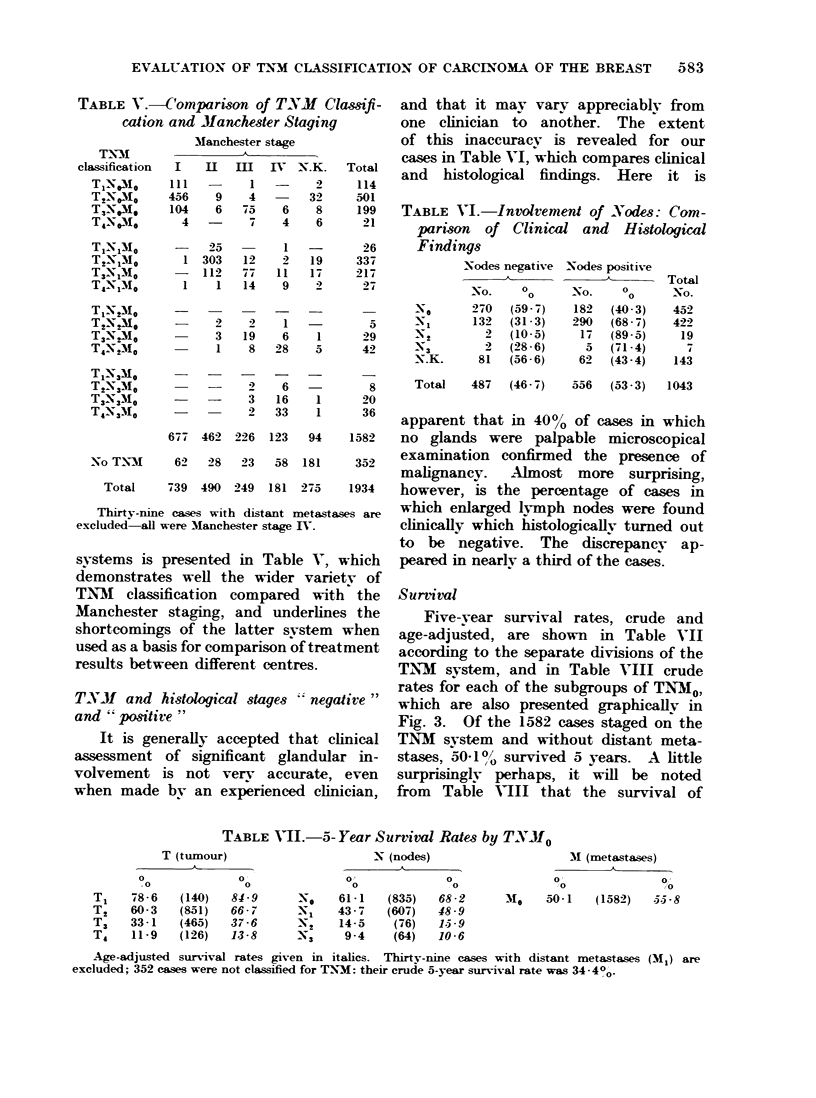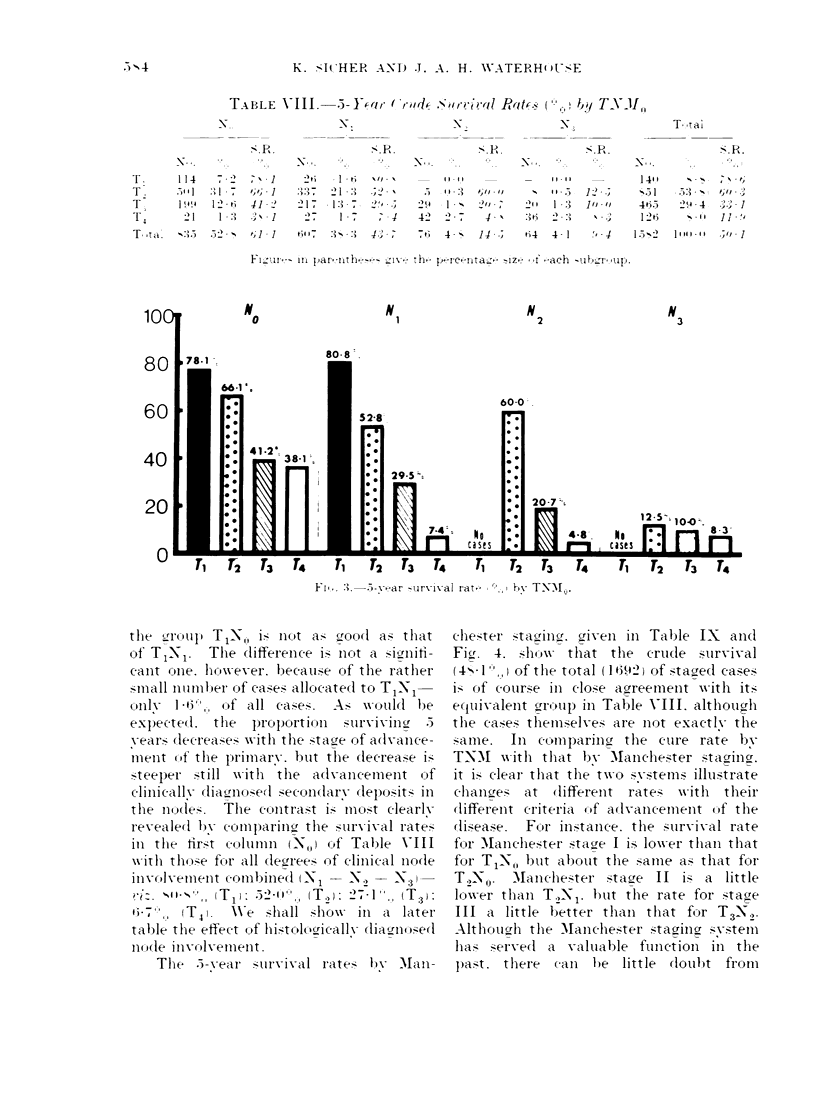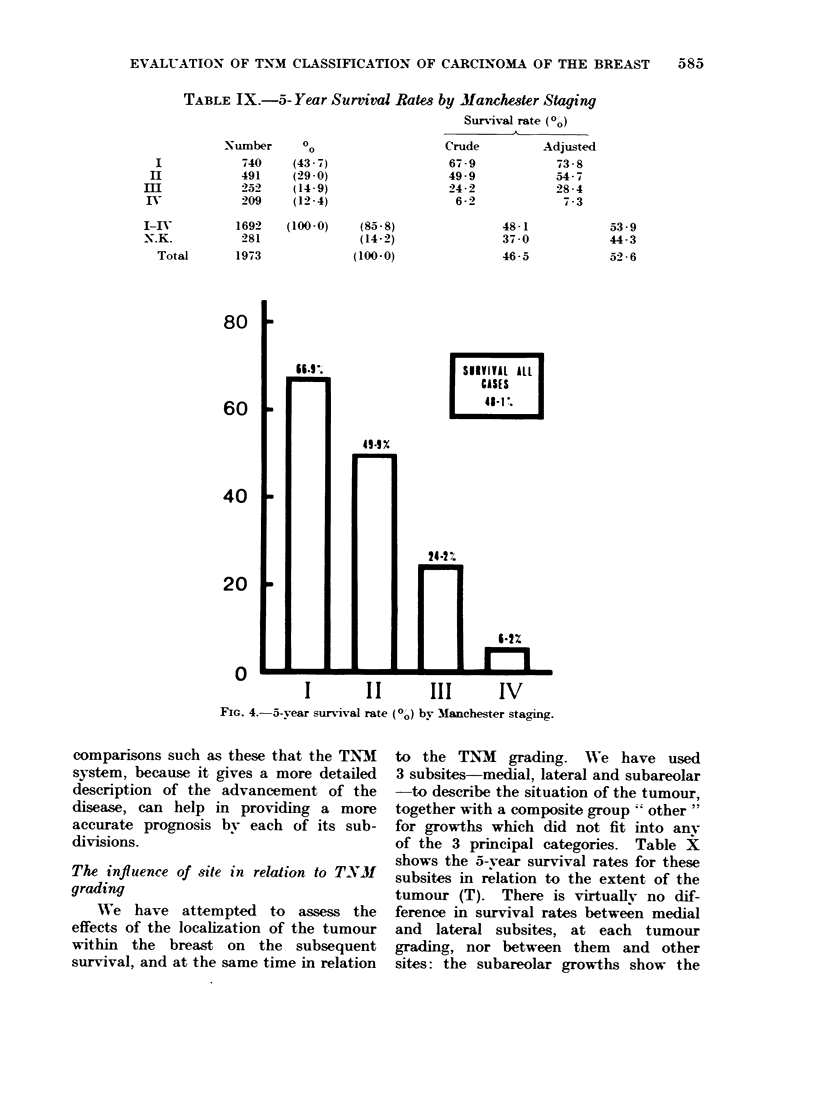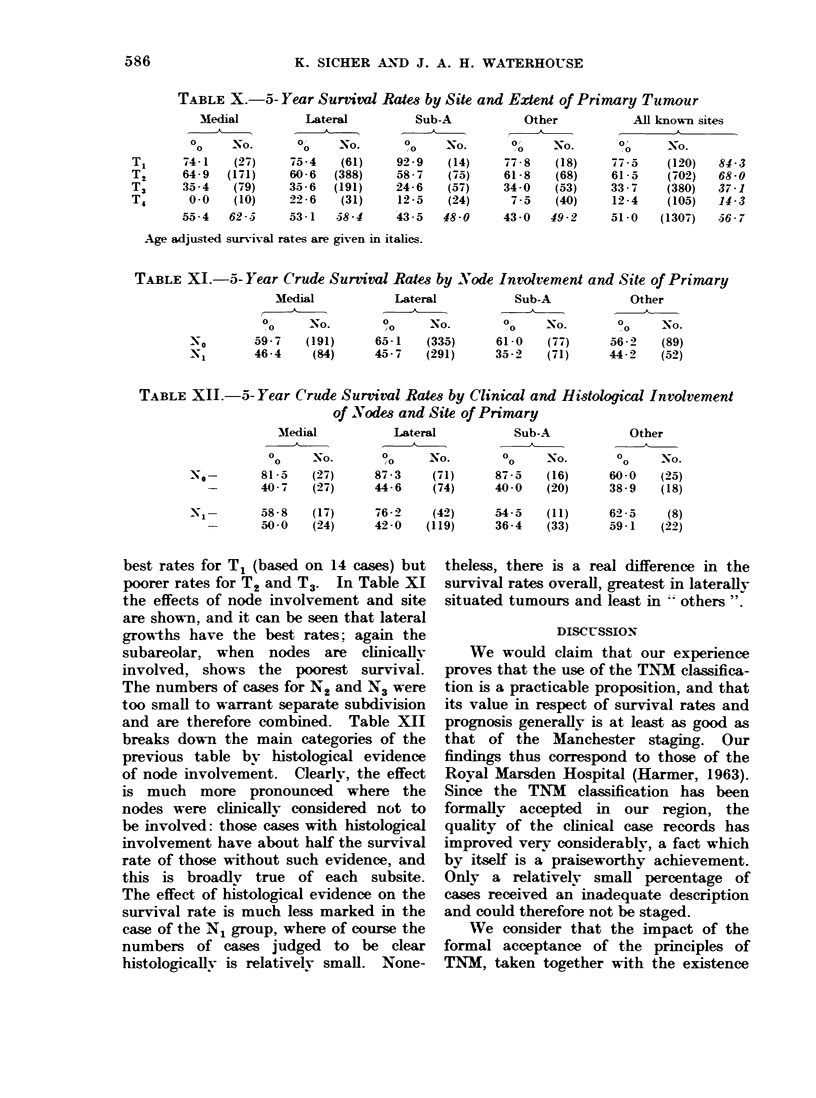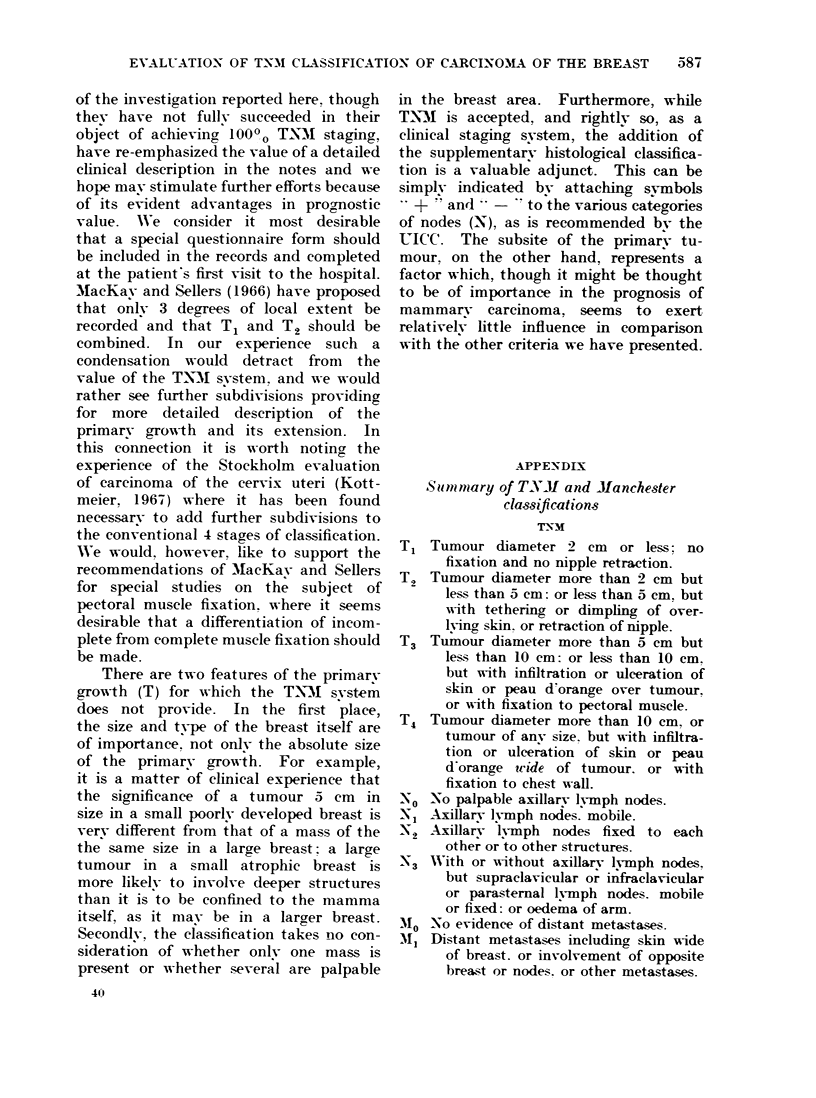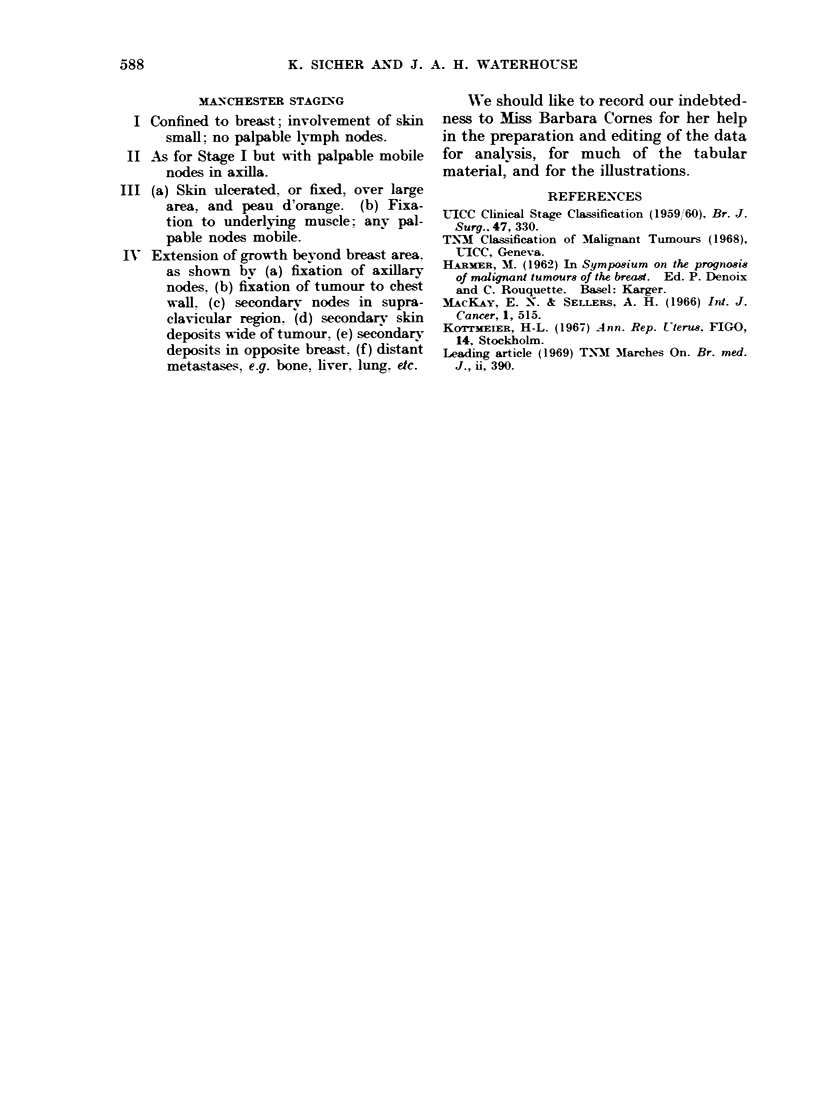# Evaluation of TNM Classification of Carcinoma of the Breast

**DOI:** 10.1038/bjc.1973.189

**Published:** 1973-12

**Authors:** K. Sicher, J. A. H. Waterhouse

## Abstract

A survey of the records of almost 2000 cases of carcinoma of the breast in the years 1960-67, since acceptance of the principles of the TNM system of classification, found only 9·2% lacking description. Although the TNM staging was not always allocated by the surgeon making the initial examination, the survey demonstrated that acceptance of the system has resulted in the inclusion of a much more detailed clinical description by the surgeon in the patient's record, from which it was generally possible to assess the TNM staging in the Radiotherapy Department. The more detailed survival rates available in the case of the TNM system, in comparison with the Manchester staging system, is cited as a further recommendation for its wider use. Although detailed localization of the tumour to subsites within the breast appears to have little influence on prognosis, the addition of a supplementary histological classification of node involvement is a valuable adjunct to the assessment of likely survival.


*Br. J. Cancer* (1973) **28**, 580

### EVALUATION OF TNM CLASSIFICATION OF CARCINOMA OF THE BREAST

K. SICHER AND J. A. H. WATERHOUSE

*From the Coventry and Warwickshire Hospital and Birmingham Regional Cancer Registry*

Received 10 July 1973. Accepted 6 September 1973

**Summary.—A survey of the records of almost 2000 cases of carcinoma of the breast in the years 1960–67, since acceptance of the principles of the TNM system of classification, found only 9·2% lacking description. Although the TNM staging was not always allocated by the surgeon making the initial examination, the survey demonstrated that acceptance of the system has resulted in the inclusion of a much more detailed clinical description by the surgeon in the patient's record, from which it was generally possible to assess the TNM staging in the Radiotherapy Department. The more detailed survival rates available in the case of the TNM system, in comparison with the Manchester staging system, is cited as a further recommendation for its wider use. Although detailed localization of the tumour to subsites within the breast appears to have little influence on prognosis, the addition of a supplementary histological classification of node involvement is a valuable adjunct to the assessment of likely survival.**

THE IMPORTANCE of clinical classification of malignant tumours is implicit in any worthwhile assessment of the relative efficacy of treatment regimens. In the field of mammary cancer in particular, much of the management of which is still a subject of controversy, evaluation of the relative merits of different methods of treatment depends essentially upon an accurate description of the initial findings in conveniently concise and readily accepted terms. Clinical trials are current in this and in other countries to test and compare alternative treatments, but the validity of their results and the relevance of comparisons between them hinge on the successful application of methods of description of the growth based on the same, generally accepted, principles for all centres. The TNM system as recommended by the International Union against Cancer (UICC) in 1959 sets out to fulfil this function and experience of its use in breast cancer is now becoming more extensive. It was recommended at the IXth International Cancer Congress in 1966 that the system should be accepted internationally, for an initial period of at least 5 years.

The principles of TNM staging of breast tumours, as described in the *British Journal of Surgery* (1960) or in the UICC's booklet (1968)* were formally accepted for implementation by both surgeons and radiotherapists in the Birmingham Region soon afterwards. This paper sets out to make a preliminary assessment of the extent to which the recommendations have been followed in two of the Hospital Groups of the region (Groups 14 and 20), and to compare it with the Manchester system of staging which had been in general use for many years. At the same time comparisons are also made of the TNM classification with histological evidence of axillary node involvement, as well as with the situation of the tumour within the breast.

* A new edition of this Booklet was produced in 1972, proposing some modifications for use in the period 1973–77.

TABLE I.—*Number of Cases Staged by Year of Registration*

| Staging | 1960 | 1961 | 1962 | 1963 | 1964 | 1965 | 1966 | 1967 | Total |
|---|---|---|---|---|---|---|---|---|---|
| TNM and Manchester | 190 | 173 | 187 | 210 | 176 | 179 | 206 | 200 | 1521 |
| TNM only | 3 | 14 | 5 | 6 | 25 | 17 | 21 | 9 | 100 |
| Manchester only | 10 | 10 | 13 | 3 | 30 | 38 | 33 | 34 | 171 |
| Neither | 14 | 31 | 34 | 25 | 15 | 21 | 17 | 24 | 181 |
| Total | 217 | 228 | 239 | 244 | 246 | 255 | 277 | 267 | 1973 |

The period of time covered is 1960–67. Table I shows that the proportion of cases staged has not altered much in this time. A specially devised form giving full details of the TNM criteria is included in the records of each case. It is made clear that the TNM staging should be made at the first examination but unhappily this condition was not invariably observed. The description of the growth, primary and secondaries, given by the surgeons was, however, sufficiently detailed in most cases for TNM assessment in the radiotherapy department. While on occasions the TNM staging is omitted, the information now given by the surgeons is much more detailed than it was before the scheme began. The simple direction "carcinoma breast—for mastectomy" has fortunately disappeared, and contrasts sharply with the present full and adequate description of the growth now given by the surgeons—an example which is followed also by their junior staff from registrars down. In nearly 2000 cases, for instance, only 9·2% were not staged at all and both TNM and Manchester systems were recorded for 77·1%.

Table II and Fig. 1 show the overall distribution by Manchester staging. Stage

TABLE II.—*Manchester Staging*

| | Number | % | % |
|---|---|---|---|
| I | 740 | 43·7 | |
| II | 491 | 29·0 | |
| III | 252 | 14·9 | |
| IV | 209 | 12·4 | |
| Total staged | 1692 | 100·0 | 85·8 |
| Not staged | 281 | | 14·2 |
| Total | 1973 | | 100·0 |

I at 43·7% is the largest group, while 14·2% had inadequate information for assessment of stage. The distribution by TNM system is shown in Table III, and in Fig. 2. As can be seen, only 2·4% had distant metastases ($M_1$).

A more detailed breakdown of the TNM cases is made in Table IV, which shows the largest single group of cases to have been classified as $T_2N_0$. A comparison of the TNM and Manchester

FIG. 1.—Distribution by Manchester staging.

FIG. 2.—Distribution by TNM categories.

TABLE III.—*TNM Classification*

| | T (tumour) | | | N (nodes) | | | M (metastases) | |
|---|---|---|---|---|---|---|---|---|
| | Number | % | | Number | % | | Number | % |
| $T_1$ | 140 | 8·7 | $N_0$ | 845 | 52·1 | $M_0$ | 1582 | 97·6 |
| $T_2$ | 858 | 52·9 | $N_1$ | 620 | 38·2 | $M_1$ | 39 | 2·4 |
| $T_3$ | 482 | 29·7 | $N_2$ | 87 | 5·4 | | | |
| $T_4$ | 141 | 8·7 | $N_3$ | 69 | 4·3 | | | |
| Total | 1621 | 100·0 | | 1621 | 100·0 | | 1621 | 100·0 |

A total of 352 cases were not staged for TNM, representing 17·8% of the total (1973 cases).

TABLE IV.—*TNM Distribution of Cases without Distant Metastases* (i.e. $M_0$ *only*)

| | $N_0$ | | $N_1$ | | $N_2$ | | $N_3$ | | Total | |
|---|---|---|---|---|---|---|---|---|---|---|
| | No. | % | No. | % | No. | % | No. | % | No. | % |
| $T_1$ | 114 | 7·2 | 26 | 1·6 | — | — | — | — | 140 | 8·8 |
| $T_2$ | 501 | 31·7 | 337 | 21·3 | 5 | 0·3 | 8 | 0·5 | 851 | 53·8 |
| $T_3$ | 199 | 12·6 | 217 | 13·7 | 29 | 1·8 | 20 | 1·3 | 465 | 29·4 |
| $T_4$ | 21 | 1·3 | 27 | 1·7 | 42 | 2·7 | 36 | 2·3 | 126 | 8·0 |
| Total | 835 | 52·8 | 607 | 38·4 | 76 | 4·8 | 64 | 4·0 | 1582 | 100·0 |

TABLE V.—*Comparison of TNM Classification and Manchester Staging*

| TNM classification | Manchester stage I | II | III | IV | N.K. | Total |
|---|---|---|---|---|---|---|
| $T_1N_0M_0$ | 111 | — | 1 | — | 2 | 114 |
| $T_2N_0M_0$ | 456 | 9 | 4 | — | 32 | 501 |
| $T_3N_0M_0$ | 104 | 6 | 75 | 6 | 8 | 199 |
| $T_4N_0M_0$ | 4 | — | 7 | 4 | 6 | 21 |
| $T_1N_1M_0$ | — | 25 | — | 1 | — | 26 |
| $T_2N_1M_0$ | 1 | 303 | 12 | 2 | 19 | 337 |
| $T_3N_1M_0$ | — | 112 | 77 | 11 | 17 | 217 |
| $T_4N_1M_0$ | 1 | 1 | 14 | 9 | 2 | 27 |
| $T_1N_2M_0$ | — | — | — | — | — | — |
| $T_2N_2M_0$ | — | 2 | 2 | 1 | — | 5 |
| $T_3N_2M_0$ | — | 3 | 19 | 6 | 1 | 29 |
| $T_4N_2M_0$ | — | 1 | 8 | 28 | 5 | 42 |
| $T_1N_3M_0$ | — | — | — | — | — | — |
| $T_2N_3M_0$ | — | — | 2 | 6 | — | 8 |
| $T_3N_3M_0$ | — | — | 3 | 16 | 1 | 20 |
| $T_4N_3M_0$ | — | — | 2 | 33 | 1 | 36 |
| | 677 | 462 | 226 | 123 | 94 | 1582 |
| No TNM | 62 | 28 | 23 | 58 | 181 | 352 |
| Total | 739 | 490 | 249 | 181 | 275 | 1934 |

Thirty-nine cases with distant metastases are excluded—all were Manchester stage IV.

systems is presented in Table V, which demonstrates well the wider variety of TNM classification compared with the Manchester staging, and underlines the shortcomings of the latter system when used as a basis for comparison of treatment results between different centres.

### *TNM and histological stages "negative" and "positive"*

It is generally accepted that clinical assessment of significant glandular involvement is not very accurate, even when made by an experienced clinician, and that it may vary appreciably from one clinician to another. The extent of this inaccuracy is revealed for our cases in Table VI, which compares clinical and histological findings. Here it is

TABLE VI.—*Involvement of Nodes: Comparison of Clinical and Histological Findings*

| | Nodes negative No. | % | Nodes positive No. | % | Total No. |
|---|---|---|---|---|---|
| $N_0$ | 270 | (59·7) | 182 | (40·3) | 452 |
| $N_1$ | 132 | (31·3) | 290 | (68·7) | 422 |
| $N_2$ | 2 | (10·5) | 17 | (89·5) | 19 |
| $N_3$ | 2 | (28·6) | 5 | (71·4) | 7 |
| N.K. | 81 | (56·6) | 62 | (43·4) | 143 |
| Total | 487 | (46·7) | 556 | (53·3) | 1043 |

apparent that in 40% of cases in which no glands were palpable microscopical examination confirmed the presence of malignancy. Almost more surprising, however, is the percentage of cases in which enlarged lymph nodes were found clinically which histologically turned out to be negative. The discrepancy appeared in nearly a third of the cases.

### *Survival*

Five-year survival rates, crude and age-adjusted, are shown in Table VII according to the separate divisions of the TNM system, and in Table VIII crude rates for each of the subgroups of $TNM_0$, which are also presented graphically in Fig. 3. Of the 1582 cases staged on the TNM system and without distant metastases, 50·1% survived 5 years. A little surprisingly perhaps, it will be noted from Table VIII that the survival of

TABLE VII.—*5-Year Survival Rates by $TNM_0$*

| T (tumour) | % | | % | N (nodes) | % | | % | M (metastases) | % | | % |
|---|---|---|---|---|---|---|---|---|---|---|---|
| $T_1$ | 78·6 | (140) | *84·9* | $N_0$ | 61·1 | (835) | *68·2* | $M_0$ | 50·1 | (1582) | *55·8* |
| $T_2$ | 60·3 | (851) | *66·7* | $N_1$ | 43·7 | (607) | *48·9* | | | | |
| $T_3$ | 33·1 | (465) | *37·6* | $N_2$ | 14·5 | (76) | *15·9* | | | | |
| $T_4$ | 11·9 | (126) | *13·8* | $N_3$ | 9·4 | (64) | *10·6* | | | | |

Age-adjusted survival rates given in italics. Thirty-nine cases with distant metastases ($M_1$) are excluded; 352 cases were not classified for TNM: their crude 5-year survival rate was 34·4%.

TABLE VIII.—*5-Year Crude Survival Rates (%) by* $TNM_0$

| | $N_0$ | | | $N_1$ | | | $N_2$ | | | $N_3$ | | | Total | | |
|---|---|---|---|---|---|---|---|---|---|---|---|---|---|---|---|
| | No. | % | S.R. (%) | No. | % | S.R. (%) | No. | % | S.R. (%) | No. | % | S.R. (%) | No. | % | S.R. (%) |
| $T_1$ | 114 | (7·2) | *78·1* | 26 | (1·6) | *80·8* | — | (0·0) | — | — | (0·0) | — | 140 | (8·8) | *78·6* |
| $T_2$ | 501 | (31·7) | *66·1* | 337 | (21·3) | *52·8* | 5 | (0·3) | *60·0* | 8 | (0·5) | *12·5* | 851 | (53·8) | *60·3* |
| $T_3$ | 199 | (12·6) | *41·2* | 217 | (13·7) | *29·5* | 29 | (1·8) | *20·7* | 20 | (1·3) | *10·0* | 465 | (29·4) | *33·1* |
| $T_4$ | 21 | (1·3) | *38·1* | 27 | (1·7) | *7·4* | 42 | (2·7) | *4·8* | 36 | (2·3) | *8·3* | 126 | (8·0) | *11·9* |
| Total | 835 | (52·8) | *61·1* | 607 | (38·3) | *43·7* | 76 | (4·8) | *14·5* | 64 | (4·1) | *9·4* | 1582 | (100·0) | *50·1* |

Figures in parentheses give the percentage size of each subgroup.

FIG. 3.—5-year survival rate (%) by $TNM_0$.

The 5-year survival rates by Manchester staging, given in Table IX and Fig. 4, show that the crude survival (48·1%) of the total (1692) of staged cases is of course in close agreement with its equivalent group in Table VIII, although the cases themselves are not exactly the same. In comparing the cure rate by TNM with that by Manchester staging, it is clear that the two systems illustrate changes at different rates with their different criteria of advancement of the disease. For instance, the survival rate for Manchester stage I is lower than that for $T_1N_0$ but about the same as that for $T_2N_0$. Manchester stage II is a little lower than $T_2N_1$, but the rate for stage III a little better than that for $T_3N_2$. Although the Manchester staging system has served a valuable function in the past, there can be little doubt from

TABLE IX.—5-*Year Survival Rates by Manchester Staging*

| | Number | % | | Survival rate (%) Crude | Adjusted |
|---|---|---|---|---|---|
| I | 740 | (43·7) | | 67·9 | 73·8 |
| II | 491 | (29·0) | | 49·9 | 54·7 |
| III | 252 | (14·9) | | 24·2 | 28·4 |
| IV | 209 | (12·4) | | 6·2 | 7·3 |
| I–IV | 1692 | (100·0) | (85·8) | 48·1 | 53·9 |
| N.K. | 281 | | (14·2) | 37·0 | 44·3 |
| Total | 1973 | | (100·0) | 46·5 | 52·6 |

FIG. 4.—5-year survival rate (%) by Manchester staging.

comparisons such as these that the TNM system, because it gives a more detailed description of the advancement of the disease, can help in providing a more accurate prognosis by each of its subdivisions.

*The influence of site in relation to TNM grading*

We have attempted to assess the effects of the localization of the tumour within the breast on the subsequent survival, and at the same time in relation to the TNM grading. We have used 3 subsites—medial, lateral and subareolar —to describe the situation of the tumour, together with a composite group " other " for growths which did not fit into any of the 3 principal categories. Table X shows the 5-year survival rates for these subsites in relation to the extent of the tumour (T). There is virtually no difference in survival rates between medial and lateral subsites, at each tumour grading, nor between them and other sites: the subareolar growths show the

TABLE X.—5-*Year Survival Rates by Site and Extent of Primary Tumour*

| | Medial | | Lateral | | Sub-A | | Other | | All known sites | | |
|---|---|---|---|---|---|---|---|---|---|---|---|
| | % | No. | % | No. | % | No. | % | No. | % | No. | |
| $T_1$ | 74·1 | (27) | 75·4 | (61) | 92·9 | (14) | 77·8 | (18) | 77·5 | (120) | *84·3* |
| $T_2$ | 64·9 | (171) | 60·6 | (388) | 58·7 | (75) | 61·8 | (68) | 61·5 | (702) | *68·0* |
| $T_3$ | 35·4 | (79) | 35·6 | (191) | 24·6 | (57) | 34·0 | (53) | 33·7 | (380) | *37·1* |
| $T_4$ | 0·0 | (10) | 22·6 | (31) | 12·5 | (24) | 7·5 | (40) | 12·4 | (105) | *14·3* |
| | 55·4 | *62·5* | 53·1 | *58·4* | 43·5 | *48·0* | 43·0 | *49·2* | 51·0 | (1307) | *56·7* |

Age adjusted survival rates are given in italics.

TABLE XI.—5-*Year Crude Survival Rates by Node Involvement and Site of Primary*

| | Medial | | Lateral | | Sub-A | | Other | |
|---|---|---|---|---|---|---|---|---|
| | % | No. | % | No. | % | No. | % | No. |
| $N_0$ | 59·7 | (191) | 65·1 | (335) | 61·0 | (77) | 56·2 | (89) |
| $N_1$ | 46·4 | (84) | 45·7 | (291) | 35·2 | (71) | 44·2 | (52) |

TABLE XII.—5-*Year Crude Survival Rates by Clinical and Histological Involvement of Nodes and Site of Primary*

| | Medial | | Lateral | | Sub-A | | Other | |
|---|---|---|---|---|---|---|---|---|
| | % | No. | % | No. | % | No. | % | No. |
| $N_0-$ | 81·5 | (27) | 87·3 | (71) | 87·5 | (16) | 60·0 | (25) |
| $+$ | 40·7 | (27) | 44·6 | (74) | 40·0 | (20) | 38·9 | (18) |
| $N_1-$ | 58·8 | (17) | 76·2 | (42) | 54·5 | (11) | 62·5 | (8) |
| $+$ | 50·0 | (24) | 42·0 | (119) | 36·4 | (33) | 59·1 | (22) |

best rates for $T_1$ (based on 14 cases) but poorer rates for $T_2$ and $T_3$. In Table XI the effects of node involvement and site are shown, and it can be seen that lateral growths have the best rates; again the subareolar, when nodes are clinically involved, shows the poorest survival. The numbers of cases for $N_2$ and $N_3$ were too small to warrant separate subdivision and are therefore combined. Table XII breaks down the main categories of the previous table by histological evidence of node involvement. Clearly, the effect is much more pronounced where the nodes were clinically considered not to be involved: those cases with histological involvement have about half the survival rate of those without such evidence, and this is broadly true of each subsite. The effect of histological evidence on the survival rate is much less marked in the case of the $N_1$ group, where of course the numbers of cases judged to be clear histologically is relatively small. Nonetheless, there is a real difference in the survival rates overall, greatest in laterally situated tumours and least in " others ".

### DISCUSSION

We would claim that our experience proves that the use of the TNM classification is a practicable proposition, and that its value in respect of survival rates and prognosis generally is at least as good as that of the Manchester staging. Our findings thus correspond to those of the Royal Marsden Hospital (Harmer, 1963). Since the TNM classification has been formally accepted in our region, the quality of the clinical case records has improved very considerably, a fact which by itself is a praiseworthy achievement. Only a relatively small percentage of cases received an inadequate description and could therefore not be staged.

We consider that the impact of the formal acceptance of the principles of TNM, taken together with the existence of the investigation reported here, though they have not fully succeeded in their object of achieving 100% TNM staging, have re-emphasized the value of a detailed clinical description in the notes and we hope may stimulate further efforts because of its evident advantages in prognostic value. We consider it most desirable that a special questionnaire form should be included in the records and completed at the patient's first visit to the hospital. MacKay and Sellers (1966) have proposed that only 3 degrees of local extent be recorded and that $T_1$ and $T_2$ should be combined. In our experience such a condensation would detract from the value of the TNM system, and we would rather see further subdivisions providing for more detailed description of the primary growth and its extension. In this connection it is worth noting the experience of the Stockholm evaluation of carcinoma of the cervix uteri (Kottmeier, 1967) where it has been found necessary to add further subdivisions to the conventional 4 stages of classification. We would, however, like to support the recommendations of MacKay and Sellers for special studies on the subject of pectoral muscle fixation, where it seems desirable that a differentiation of incomplete from complete muscle fixation should be made.

There are two features of the primary growth (T) for which the TNM system does not provide. In the first place, the size and type of the breast itself are of importance, not only the absolute size of the primary growth. For example, it is a matter of clinical experience that the significance of a tumour 5 cm in size in a small poorly developed breast is very different from that of a mass of the the same size in a large breast; a large tumour in a small atrophic breast is more likely to involve deeper structures than it is to be confined to the mamma itself, as it may be in a larger breast. Secondly, the classification takes no consideration of whether only one mass is present or whether several are palpable in the breast area. Furthermore, while TNM is accepted, and rightly so, as a clinical staging system, the addition of the supplementary histological classification is a valuable adjunct. This can be simply indicated by attaching symbols "+" and "−" to the various categories of nodes (N), as is recommended by the UICC. The subsite of the primary tumour, on the other hand, represents a factor which, though it might be thought to be of importance in the prognosis of mammary carcinoma, seems to exert relatively little influence in comparison with the other criteria we have presented.

### APPENDIX

### *Summary of TNM and Manchester classifications*

#### TNM

$T_1$ Tumour diameter 2 cm or less; no fixation and no nipple retraction.

$T_2$ Tumour diameter more than 2 cm but less than 5 cm; or less than 5 cm, but with tethering or dimpling of overlying skin, or retraction of nipple.

$T_3$ Tumour diameter more than 5 cm but less than 10 cm; or less than 10 cm, but with infiltration or ulceration of skin or peau d'orange over tumour, or with fixation to pectoral muscle.

$T_4$ Tumour diameter more than 10 cm, or tumour of any size, but with infiltration or ulceration of skin or peau d'orange *wide* of tumour, or with fixation to chest wall.

$N_0$ No palpable axillary lymph nodes.

$N_1$ Axillary lymph nodes, mobile.

$N_2$ Axillary lymph nodes fixed to each other or to other structures.

$N_3$ With or without axillary lymph nodes, but supraclavicular or infraclavicular or parasternal lymph nodes, mobile or fixed; or oedema of arm.

$M_0$ No evidence of distant metastases.

$M_1$ Distant metastases including skin wide of breast, or involvement of opposite breast or nodes, or other metastases.

MANCHESTER STAGING

I Confined to breast; involvement of skin small; no palpable lymph nodes.

II As for Stage I but with palpable mobile nodes in axilla.

III (a) Skin ulcerated, or fixed, over large area, and peau d'orange. (b) Fixation to underlying muscle; any palpable nodes mobile.

IV Extension of growth beyond breast area, as shown by (a) fixation of axillary nodes, (b) fixation of tumour to chest wall, (c) secondary nodes in supraclavicular region, (d) secondary skin deposits wide of tumour, (e) secondary deposits in opposite breast, (f) distant metastases, *e.g.* bone, liver, lung, *etc.*

We should like to record our indebtedness to Miss Barbara Cornes for her help in the preparation and editing of the data for analysis, for much of the tabular material, and for the illustrations.

REFERENCES

UICC Clinical Stage Classification (1959/60), *Br. J. Surg.*, **47**, 330.

TNM Classification of Malignant Tumours (1968), UICC, Geneva.

HARMER, M. (1962) In *Symposium on the prognosis of malignant tumours of the breast*. Ed. P. Denoix and C. Rouquette. Basel: Karger.

MACKAY, E. N. & SELLERS, A. H. (1966) *Int. J. Cancer*, **1**, 515.

KOTTMEIER, H-L. (1967) *Ann. Rep. Uterus*, FIGO, **14**, Stockholm.

Leading article (1969) TNM Marches On. *Br. med. J.*, ii, 390.